# Learning Platforms for Implementing Formative Interventions to Promote the Health and Safety of Workers in Brazil

**DOI:** 10.3389/fpsyg.2020.619593

**Published:** 2021-02-17

**Authors:** Manoela Gomes Reis Lopes, Rodolfo Andrade de Gouveia Vilela, Amanda Aparecida Silva-Macaia, Vinícius Monteiro de Paula Guirado, Marco Antonio Pereira Querol

**Affiliations:** ^1^Department of Community Medicine, Federal University of Piauí, Teresina, Brazil; ^2^Department of Environmental Health, School of Public Health, University of São Paulo, São Paulo, Brazil; ^3^Division of Neurosurgery, Hospital das Clínicas, São Paulo Medical School, University of São Paulo, São Paulo, Brazil; ^4^Division of Neurosurgery, Santa Casa de São Paulo School of Medical Sciences, São Paulo, Brazil; ^5^Department of Agronomic Engineering, Federal University of Sergipe, São Cristóvão, Brazil

**Keywords:** organizational learning, learning platform, Change Laboratory, interventionist, activity system, Cultural-Historical Activity Theory

## Abstract

Formative intervention methodologies, such as the Change Laboratory (CL), are increasingly being used in work environments. However, the learning process entailed in the application of these methodologies has received insufficient attention and may be facilitated through the use of learning platforms. We examined the development of learning and training strategies for implementing formative interventions, drawing on the experiences of a research group focusing on workers’ health. Information obtained from individuals involved in CL formative activities was analyzed and interpreted using Cultural-Historical Activity Theory and the theory of expansive learning. The process of learning to implement formative interventions unfolded gradually, beginning with the interventionists’ initial exposure to abstract concepts that they subsequently internalized via various mediations and applied in concrete situations. Four key interventionist training strategies used to foster collective learning were identified: (1) promoting dialogues and exchange of experiences, (2) creating environments for continuous learning and permanent discussion (seminars and post-graduate courses and the use of communication technologies), (3) creating spaces for experimentation and the practical application of concepts (case studies and participation in interventions), and (4) the use of the double stimulation method during training programs.

## Introduction

Societal, technological, and market transformations have changed contemporary work environments, and corresponding methods of coordinating and regulating work. These changes, in turn, have affected occupational health, notably occupational diseases and accidents among employees. To ensure short-term profits and meet their investors’ expectations of returns, corporations operating in a financialized environment adopt aggressive and sometimes openly violent management strategies based on goals and performance indicators that affect the quality and reliability of the work, lives, and health of workers (Muller, 2018; Lima and Dias, 2020).

Historically, the approach for safeguarding workers’ health within the Brazilian Unified Health System (SUS)^1^ represents a critique of the factorial view. This view is associated with approaches to hygiene, safety, and occupational health that do not consider the work process in its totality or the systemic interactions that could explain accidents and occupational diseases (Dias, 1994; Minayo-Gomez and Thedim-Costa, 1997).

This approach, which represents a shift from earlier paradigms, concepts, and practices, seeks to encourage the production of autonomy through the construction of social networks and workers’ participation in health planning and management. Accordingly, it entails the use of a transdisciplinary intersectoral approach that is holistic and views participating workers as subjects and partners capable of contributing their own knowledge to deepen understanding of the impacts of work on the health-disease process. More broadly, it is aimed at developing effective interventions for transforming society (Dias, 1994; Minayo-Gomez and Thedim-Costa, 1997). In this historical context, PesquisAT was established as a research and intervention group working to promote and protect workers’ health, prevent occupational diseases, and transform their working conditions. This research group has worked in the area of workers’ health for the past 8 years.

In this paper, we argue that interventions grounded in systemic approaches that promote social as well as expansive learning should be developed to deal with the complexities and challenges resulting from these transformations. The majority of interventions in work environments are based on the principle of a specific but generic solution implemented locally. However, situations are proliferating in which such ready-made solutions are inadequate for solving a crisis relating to a work activity, thus requiring the formulation of new principles (Bodrožiæ, 2008; Bodrožiæ and Adler, 2018), particularly when dealing with complex situations.

Moreover, contemporary societal challenges require interventions that promote social learning (Wals, 2007). Theories on learning processes that have evolved in the long run are increasingly more socially rather than individually oriented (Paavola et al., 2004; Blackmore, 2007). An increasing number of studies have focused on learning and innovation, entailing a collective process through which individuals create new principles for guiding activities in a crisis. One way of promoting collective learning is through formative interventions (Engeström et al., 2014; Laitinen et al., 2016; Sannino et al., 2016). A formative intervention can be understood as an intervention in which facilitators offer participants resources that enable them to design and implement practical experiments that can lead to radical expansive innovations that can be applied to tackle contradictions, historically feeding into the goods and services production model (Sannino et al., 2016).

These development interventions promote expansive learning (Engeström, 2011) and transformative agency (Vänninen et al., 2015; Lopes et al., 2018), both of which can be fostered using the Change Laboratory (CL) method. Expansive learning takes place when the object/purpose of an activity is qualitatively transformed so that contradiction within and between the elements of the activity are resolved (Engeström, 2001). Expansive learning can be purposefully stimulated by fostering transformative agency, which is understood as intentional collective and individual actions aimed at transforming an activity (Haapasaari et al., 2014). The CL method is based on Cultural-Historical Activity Theory (CHAT), and it was developed by Finnish researchers. The CL application intends to improve and promote the expansive learning on work activities, using the double stimulation method (Virkkunen and Newnham, 2013). This method was proposed by Vygotsky (Engeström, 2007), and two sets of stimuli are presented to the subject: the first stimulus refers to objects of his activity and the second stimulus can be signs, artifacts or concepts. This double stimulus helps the subjects to organize the activity and thus the development of empowerment (Vygotsky, 1998; Engeström, 2007).

Change Laboratory and CHAT appear to offer solutions to these challenges in the area of health promotion and accident prevention (Vilela et al., 2020c). Interventions in this area usually produce good diagnostics but focus too closely on perceived problems while disregarding the historical development of the concerned activity and the collective and organizational dimensions of learning that are required to sustain the innovations. However, to master new theoretical and methodological tools, such as CL and CHAT, a set of actions needs to be developed for the intervention team, which is the object of investigation in this study. PesquisAT chose to apply CHAT and CL because the approaches previously used, while contemplating a systemic vision, did not offer analytical and pedagogical tools for promoting organizational and participants’ learning during the intervention process. In section “PesquisAT’s Trajectory Prior to the Emergence of the Learning Platform for Formative Interventions” we present in more detail the context that led to PesquisAT’s adoption of the CL methodology.

Change Laboratory is a formative intervention methodology that provides the basis for collaborative planning of innovations within a variety of organizational environments achieved through expansive learning (Engeström et al., 1996; Sannino and Engeström, 2017). Increasing interactions and the ability to achieve collective transformations, which are essential for successfully implementing CL, depend on the translation of knowledge mediated by the team of mentors. The training and qualification of the participants is also crucial to the success of the interventions (Querol et al., 2019; Hurtado et al., 2020; Vilela et al., 2020c), which could be considered as a learning platform.

Röling (1994) proposed the notion of platforms as a means of supporting collective action. His concept was based on the notion of knowledge systems composed of “the articulate group of actors, networks and/or organizations, supposed to work synergistically in order to support knowledge processes that improve the correspondence between knowledge and environment, and/or the control given by the use of technology in a certain domain of human activity” (Röling, 1992, p. 48).

More recently, Le Masson et al. (2009) proposed the idea of platforms for designing platforms, that is, a learning space for constructing other activities. According to these authors, the cooperative designing of a platform entails three main processes: (1) managing value and product creation; (2) organizing production and knowledge acquisition with partners, offering support for experimentation, and using specific knowledge production devices; and (3) managing partners’ interests and creating value for them and for their industry. In spite of these authors’ important contributions, their discussion of the operating processes of these design platforms (e.g., the collaborative spaces for design) remains confined to theory, making it difficult to understand the connections among different elements, such as value, partners, and their primary activities.

According to this conception, a formative intervention is itself a design platform. The implementation of formative interventions requires multiple learning platforms, including one for training the interventionist. A learning platform is a learning activity, which like any other activity is mediated by instruments, rules, a division of labor, and change agents that collaboratively transform another activity or network of activities. The learning process entailed in a formative intervention requires not only learning during the intervention but also the creation of platforms as spaces where learning continues after the interventionist project ends.

The implementation and use of formative interventionist methodologies entails complex theoretical devices and requires an understanding of interventionist concepts, theories, and principles (Virkkunen and Newnham, 2013) as well as theoretical and practical proficiency in the targeted field of the intervention.

Interventionist researchers learn how to use tools such as models, analytical concepts, and methods during their training as well as to create networks and participate in experiments. Interventionists have to cultivate skills that include negotiating with the managers of an organization, collecting and analyzing preliminary data so that they can formulate a contrasting hypothesis, and analyzing data produced during the expansive learning cycle that unfolds during the intervention (Virkkunen and Newnham, 2013).

Depending on the country, for example in Brazil, the CL is not widely known and could be characterized as a paradigm shift in the intervention methodologies concerning the work process which presupposes protagonism of the organization’s actors for the redesign of the activity system (AS). In addition, in order to obtain good results, during the CL application it is important that the interventionist-researcher has mastery of the method so that he can perform better the ethnographic data collection and elaborate hypotheses about the main historical contradictions. These steps will help during CL conduction, as well as in the choice of mirror data which reflects the activity’ reality, and will be used for the historical and systemic analysis of the main problems experienced in the organization, and thereby provide expansive learning through the involvement and emotional commitment of the participants (Virkkunen and Newnham, 2013; Vilela et al., 2020c).

Recent studies have shown that permanent learning can be considerably improved by providing professionals with learning tools and creating social spaces for learning (Haapasaari and Kerosuo, 2015; Haapasaari et al., 2018).

The learning process experienced by CL participants has similarities to the concept of permanent health education developed by educators who have been working to strengthen Brazil’s Unified Health System (SUS). The concept of permanent health education guides the daily learning process and entails a commitment to the collective, where the actors are the main decision makers regarding their own work-related activities. According to this concept, within the art of acknowledging the country’s diversity and plurality, daily life is considered a place of invention and of embracing challenges and creatively replacing models through the application of cooperative, collaborative, integrated, and courageous practices. This concept is guided by transformative formative practices, commencing with a collective analysis of work processes within a local setting. Activities are designed in a bottom-up manner, entailing critical analysis and experience sharing and articulating with productive activities that encompass production, management, education, and social control. Permanent follow-up and technical support are also envisaged, and practices are multifactorial, incorporating knowledge, values, power relations, and the work organization. Significant learning occurs through working with elements that “make sense” for the concerned subjects (significant learning) (Ceccim, 2005; Ceccim and Feria, 2009; Ministério da Saúde Brasil, 2014).

Despite these advances, little is known about the structure (mediators) and functioning (actions) within these social spaces that are required for the continuation of expansive learning after interventionist projects conclude.

In this study, we argue that learning platforms that combine spaces for emotional engagement and concrete tools can facilitate the learning process relating to formative intervention methodologies and their adaptation to local conditions and the development of ASs within an intervention. An understanding of students’ learning processes and professionals’ pedagogical activities aimed at training mediators for this type of intervention may promote long-term assessments as well as opportunities for continuous improvement of instructors’ knowledge (Arkoudis et al., 2013). The findings of a systematic review study indicated that Kirkpatrick’s four-level training evaluation model could be appropriate for this purpose (Söderlund et al., 2011). This model, which is a pedagogical performance assessment tool (Kirkpatrick and Kayser-Kirkpatrick, 2014), has been applied in the fields of business (Han and Boulay, 2013), healthcare (Carlfjord et al., 2017), and, more recently, higher education (Praslova, 2010; Taras et al., 2013).

However, little is known about the process entailed in this specific learning activity, namely platform-based learning for implementing formative interventions. This study intends to answer the following question. How can interventionist researchers be prepared to perform formative interventions in the area of workers’ health? Accordingly, the study aims at evaluating the training strategies for preparing professionals, researchers, and students to implement CL interventions, drawing on the experiences of PesquisAT.

## Materials and Methods

We conducted a case study for evaluating the learning process experienced by CL participants trained by PesquisAT, and invited attendees of any training and learning event organized by PesquisAT during the period 2012 to August 2020 to participate. The study covered all of the theoretical or practical activities (post-graduate courses and extracurricular courses, training workshops, study groups, and CL seminars) entailed in the CL training and learning method offered by PesquisAT.

We collected quantitative and qualitative data to ensure complementarity between the methods and the validation of results achieved through a process of triangulation (Denzin, 1970; Reeves et al., 2013). Data were collected online through two different phases using the following strategies: (1) semi-structured interviews (qualitative data) and (2) questionnaires designed as Google forms® specifically for this study (quantitative and qualitative data).

The semi-structured interviews were recorded, lasted around 1 h and included all PesquisAT members who were directly working or were involved with activities related to CHAT and CL from the beginning. Eight researchers with different backgrounds (engineers, medical doctors, physiotherapists, agronomist, social scientists, and psychologists) were interviewed. The interviewee played different roles at PesquisAT group in 2012 as researchers, professors, post-doctoral, and Ph.D. position. The objective of these interviews was to grasp the history and motivations behind the use of this method by eliciting a narrative on how and when they learned the method and what activities were organized to improve professionals’ training experiences. In this study, we applied the AS model proposed by Engeström (1987, 2016) as an analytical tool to examine the structure and changes in the activities of learning the CL method. At the onset, the purpose of the survey was briefly described, followed by the presentation of content relating to the informed consent form. After reading the terms, the participants were asked whether or not they agreed to participate in the survey.

The form was divided into three sections comprising open-ended and multiple choice questions about learning and training processes relating to CL, teaching materials used, adverse effects of training activities, suggested improvements, learning CHAT concepts, and perceived difficulties during CL application. The open-ended questions provided qualitative data, and the multiple choice questions provided quantitative data. The first section was generic (e.g., name, email, age, level of education), the second section focused on the learning process, and the third section was devoted to CL concepts and principles.

The design of the questionnaire was inspired by the Kirkpatrick and Kirkpatrick (2006) model, with its questions addressing levels two to four of that model, which has been extensively reviewed in the context of the fifty-year celebrations marking its inception (Kirkpatrick and Kayser-Kirkpatrick, 2014). It comprises four evaluation levels with the following analytical components: (1) reactions (whether participants responded favorably to the training or intervention), (2) learning (whether participants acquired the intended knowledge, skills, or attitudes based on their participation in the training event or intervention), (3) behavior (the degree to which participants subsequently changed their behaviors in other settings, such as the workplace, after participating in the training program or intervention), and (4) results (whether the overall objectives were achieved in relation to the anticipated long-term outcomes as a result of the interventions and subsequent reinforcement) (Kirkpatrick and Kayser-Kirkpatrick, 2014). We did not include level one in our questionnaire because the participants included individuals who may have participated in a training action some time back, whereas the evaluation of their reactions would be more effective for events occurring after shorter intervals.

The questionnaire was emailed to a total of 133 individuals who participated in CL training activities promoted by PesquisAT from January 2012 to August 2020. The participants were formally enrolled in post-graduate courses and/or summer courses, CL seminars, and CL workshops under the ITAPAR project^2^. A reminder was sent to participants 3 days before the deadline for receiving the questionnaire set by the researchers. As only 13 emails were undelivered and mails returned to sender, a new email search was initiated and a reminder to complete the form was sent out. A total of 39 people responded, which corresponds to 29.3% of the invited participants.

The study had a small sample with eight interviews and 39 questionnaires. Even if eight interviews were conducted, it is important to mention that we interviewed all the PesquisAT members who were directly working or involved with activities related to CHAT and CL from the beginning. About the 39 questionnaires, which correspond to 29.3% of the invited participants, it could be an important limitation and bias for the obtained results because they mostly represent the people who carry out activities with the research group currently. We believe that a long time passed from the first courses which could have had an impact on this number, because most of the invited participants were not participating in the post-graduate program and could not have recognized the sender of the e-mail. Furthermore, the email with the questionnaire link was sent by one researcher who is not known by most of the invited participants what could explain the low tax of response. The data collection was made during the Sars-Cov-2 pandemic, a moment that the contact was mainly by virtual way, and people were receiving many emails, which could also have influenced this number. We believe that beside these limitations, the study had strengths to consider. The main strength is the long time period covered, which is not so common and gives an idea from all the process. Another important strength is the focus on a real case study which enriches the analysis with multiple perspectives from the learning process. Besides that, we believe the use of mixed methods helps to analyze better the case. Considering these aspects, we believe the study has important contributions for the learning platforms of formative interventions and the results could be generalized to other areas besides workers’ health.

### Data Analysis

All the interviews were transcribed, and after reading the transcripts and the responses to open-ended items in the questionnaire, we codified the text into actions related to “learning the CL method,” highlighting the interviewees’ reflections about each action. We also extracted references to motivations for learning. The data was organized to identify events related to learning the CL and the evaluation that the interviewees made of them; that is, it was identified how the interviewee learned the method and how he/she was involved from the beginning with the training of other people. Analyzing the events over time, made it possible to understand how and when the CL became an instrument and an object for PesquisAT, in addition to the influence on the training of professionals to be interventionists in the method.

From this first organization, it was possible to analyze common events and differences among narratives, in order to constitute not only a sequence of events, but also to qualify them and recognize what would be the critical events (Sewell, 1996). We listed and collectively discussed all of the actions and accordingly constructed a timeline of training events relating to the CL method organized by PesquisAT. In light of our analysis of the timeline, we constructed a narrative, identifying critical events in the group’s history and construction of training ASs before and after the introduction of CL. Critical events can be understood as a sequence of events that determine more radical transformations (Sewell, 1996) of some of the AS elements. Consequently, we were able to grasp historical contradictions in the training activities among the different actors involved. We also analyzed the learning platform, especially the learning, behavior, and results levels in the Kirkpatrick and Kayser-Kirkpatrick (2014) model, using data extracted from the questionnaires.

We applied the principles of CHAT and the theory of expansive learning, which are the basis of CL (Engeström, 1987, 2016), as conceptual tools in the analysis. CL combines five principles derived from the two aforementioned theories: cultural mediation of human actions theory, historicity, multivoicedness, contradiction as a source of change and development, and the possibility of expansive transformations within an AS (Engeström, 2001).

For the quantitative data, we analyzed them also in the light of qualitative data. Moreover, we made a descriptive analysis by calculating the frequency of the responses in percentage, which helps to understand the data considering the total of them. Moreover, the quantitative analysis helped to quantify the perception pointed out by the interviewee.

This study was conducted under the Innovation and Transformation for Prevention Activity of Professional Risks (ITAPAR) Research Project (FAPESP 2019/13525-0) and was approved by the research ethics committee of the School of Public Health at the University of São Paulo under CAAE protocol 36516620.6.0000.5421.

## Pesquisat’s Trajectory Prior to the Emergence of the Learning Platform for Formative Interventions

In this section we present the context that led to PesquisAT’s adoption of the CL methodology and the emergence of a learning platform for training interventionists to conduct formative interventions.

The learning platform for training interventionists is an activity that has as its object the individual or collective subject of another activity that in this case is a CL subject. The CL is itself an activity of learning and developing one or more other activities, for example, productive activities.

**FIGURE 1:**
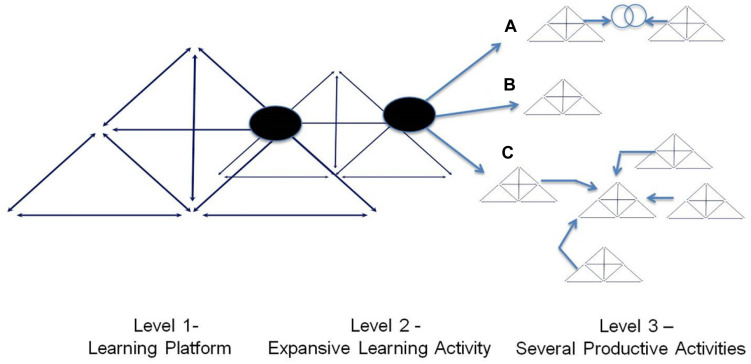
The relationship between the learning platform and expansive learning activity: Applying the Change Laboratory method to productive activity. **(A)** Change Laboratory (CL) boundary crossing, **(B)** CL within single activity systems, and **(C)** CL within a network of activity systems.

The relationships among these activities, comprising three levels, are depicted diagrammatically in Figure 1. The first level of activity, understood here as a training activity, entails training the interventionist. The second level comprises the formative intervention activity, that is, the CL, in which the interventionist mediates expansive learning actions. The third level comprises productive activity, focusing on problems relating to work processes that lead to accidents or health problems, analyzed from the perspective of occupational safety and health. Each of these levels entails connected rules, a division of labor, subjects, objects, instruments, and communities that are connected with each other, forming an AS, represented graphically within a triangle (Leontiev, 1978, 1981; Engeström, 1987, 2016).

We applied this model within a case study of the PesquisAT group’s experiences in conducting CL training from 2012 to 2020. Considering different levels of activities depicted in Figure 1, the object of the group’s productive activity would be the protection of workers and promoting their health as well as improving working conditions. Because this model is dynamic, changes occurring at one level can induce changes in other levels. In other words, a need or change in any element at the level of productive activity could lead to changes in the first and/or second levels Similarly, alterations in the PesquisAT learning platform and/or CL formative intervention could lead to alterations in other levels.

All three levels are interconnected and their interplay is dialectical. That is, each time the interventionist participates in a new activity of the formative intervention, he or she learns and practices new concepts and strategies that can be incorporated into future interventions. At the same time, more actions to support learning, or even the learning platform, become necessary. Consequently, interventionists’ capabilities keep increasing, and they can become the subject at the learning platform, fostering new interventionists as we will subsequently show.

PesquisAT was formally established in 2007. Initially, its headquarters were located at the Workers’ Health Reference Centre (CEREST^3^) in Piracicaba, a city in the state of São Paulo in Brazil. PesquisAT was created as a collaborative forum by professionals working in the health surveillance service of SUS, researchers from Fundação Jorge Duprat Figueiredo, de Segurança e Medicina do Trabalho (Fundacentro^4^), professors from various universities, personnel from the Labor Prosecutor Office, fiscal auditors from the Ministry of Labor and Employment, and post-graduate students (Vilela et al., 2020a). It has continuously expanded by welcoming new members from other institutions.

The group is currently composed of professionals from diverse fields, making it multi- and interdisciplinary. They include engineers, safety technicians, medical doctors, nurses, physiotherapists, occupational therapists, social scientists, lawyers, and psychologists.

Since its foundation, the group has sought to integrate theory and practice within its research projects. Examples of such projects include the development of SIVAT Piracicaba, which is Piracicaba’s work accident surveillance system; an information facility on health and safety indicators available to the community; the development of a Model of Analysis and Prevention of Accidents (MAPA); the creation and maintenance of the Accidents at Work Forum (FórumAT), and the promotion of an interdisciplinary, intersectoral, and dialogical approach involving different actors and institutions (Vilela et al., 2020a).

Guidelines developed for the surveillance and prevention of work-related risks indicate that actions should not comprise punctual, isolated inspections; rather, the objective should be broadened to include the resources mobilized, the actors involved, and the spaces where actions occur (Ministério da Saúde Brasil, 2012). Consequently, over time, the group has sought to introduce new qualifications and methodologies that could facilitate workers’ health surveillance, such as incorporating concepts derived from activity ergonomics (Guérin et al., 2004).

**FIGURE 2:**
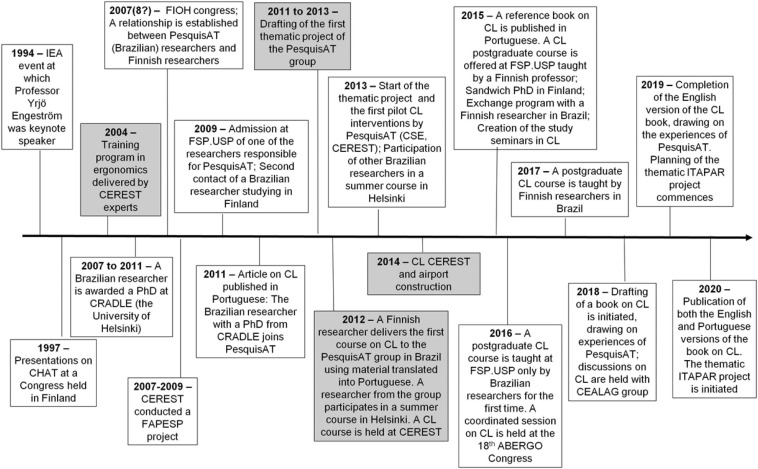
Timeline of critical events pertaining to the consolidation of PesquisAT. Critical events for PesquisAT are shaded gray. IEA, International Ergonomics Association; CHAT, Cultural-Historical Activity Theory; CEREST, Workers’ Health Reference Centre; CRADLE, Centre for Research on Activity, Development and Learning; FIOH, Finnish Institute of Occupational Health; FAPESP, São Paulo Research Foundation; FSP.USP, School of Public Health, University of São Paulo; CL, Change Laboratory; CSE, School Health Centre; ABERGO, Brazilian Ergonomics Association; CEALAG (CEALAG is a non-profit civil association founded by professors based at the Collective Health Department of the Santa Casa de São Paulo School of Medical Sciences, which focuses on the development of the health sciences. Its specific activities are research, technical support, training and accreditation, advisory services in the management of health services, and the promotion of health-related social assistance.), Centre for Studies Augusto Leopoldo Ayrosa Galvão; ITAPAR, Innovation and Transformation for Prevention Activity of Professional Risks.

In 1997, an intervention to check compliance with health and safety norms was implemented in a meat-processing plant, which had a high rate of work accidents. In spite of the company’s compliance with all of the requirements of public agencies, its accident rate remained high in 2008 (Vilela et al., 2012). In 2004, members of the group that would later become PesquisAT attended a training program on activity ergonomics (Figure 2), which broadened their understanding of the organizational causes of accidents and influenced the development of the CL training. This program is thus considered a critical event in the timeline of CL development.

Four critical events stand out in the history of PesquisAT (Figure 2): the ergonomics training conducted in 2004, the development of a thematic project from 2011 onward, international exchanges commencing from 2012, and the first CL interventions in 2014. These events prompted more radical changes in the learning platform and in the AS of the CL formative intervention, generating needs, contradictions, and expansion within the ASs.

The ergonomics training course (Guérin et al., 2004; Vilela et al., 2014) can be considered the first critical event in PesquisAT’s trajectory toward creating interventionists (Figure 2). Both of its activities–performing interventions and training new interventionists focusing on working conditions–changed when different elements within an AS changed through the incorporation of new instruments, rules, and changes in the division of labor (Vilela et al., 2018). Many of these elements, such as the reconciliation of theory and practice, were aligned with CHAT and with Permanent Health Education. Conceiving the learning process as a spiral process, we posit that the new elements served as germinal cells for further CHAT training.

The relationship between the training course in ergonomics and the CL post-graduate course developed years later by PesquisAT is mentioned in the following excerpt from an interview:

“What we did with CL was close to that approach [in the ergonomics course conducted in 2004]. The student learns by testing concepts in practical situations. We did that in class using exercises; that was the idea in that training.” (Interviewee 5).

**FIGURE 3:**
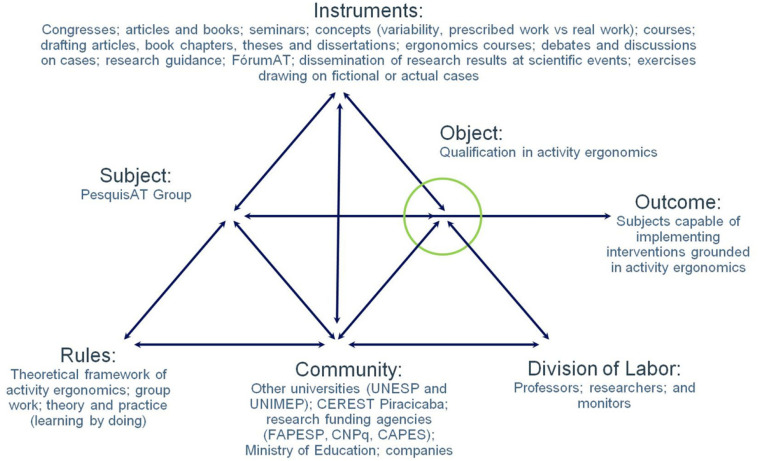
PesquisAT’s pre-Change Laboratory learning platform (2004).

Following the ergonomics course held in 2004, members of PesquisAT began to use concepts derived from activity ergonomics (Figure 3). In that course, which adopted an approach that combined theory and practice, the students had to analyze actual cases to pass. Discussions about the cases were mediated by mentors to facilitate the learning process. As noted above, this format influenced many researchers, who started applying these strategies in their classes on returning to their academic environments.

As depicted in Figure 3, as a rule, the theoretical and practical characteristics of the approach are grounded in the principle of “learning by doing” that is subsequently reinforced by other elements of the CL training activities.

“[Interviewee 5] mentioned that we used the ‘learning by doing’ approach in our practices at CEREST; one doesn’t have to become a specialist first and act later. I think that it was learning by doing.” (Interviewee eight speaking about the pre-CL period).

The experiences acquired through the ergonomics course also contributed to collective learning. As depicted in Figure 3 under “AS instruments,” the principle of “dialogical interaction” was pointed out as collective debates and reflections as well as during students’ participation as mentors or monitors, as noted under the “division of labor” category. In the process of dialogical interaction, knowledge is internalized and subsequently externalized and socialized through a process that differs from one associated with lectures and didactic books that configure expositive and unidirectional strategies. During the socialization process, the course participants exchanged experiences, which contributed to the learning process.

In this pre-CL phase, mediators also played a role in organizing collective learning, thus deepening ergonomics-based knowledge. Mediators comprise models, signs, and tools for subjects that mediate the subject’s actions in relation to the object (Cole, 1998). Under the category of “instruments” in Figure 3, commonly applied mediators were various exercises drawing on real and/or fictitious cases that served as a basis for discussions on ergonomic concepts relating to the activity. These mediators can mobilize the daily experiences of the apprentices for use in case analyses, enabling associated concepts to be clearly discerned. Similarly, other mediators were used in the CL learning platform for formative interventions.

The theoretical framework of the activity ergonomics course formed the basis of a new study on the meat-processing plant, described earlier, conducted under a project implemented in 2008 and financed by the São Paulo Research Foundation (FAPESP)^5^. The study enabled the identification of previously unexplored organizational factors associated with accident causation (Vilela et al., 2012). However, suggestions regarding organizational causes were not incorporated into the company’s operations. This example of interventionist research in the meat-processing plant highlights a situation that is prevalent within this field of work. For example, the researchers’ recommendations are not accepted by the companies, and attempts to transform working conditions prove difficult, particularly when related to organizational causes that are associated with the greatest danger for the occurrence of illnesses or accidents.

It was evident that PesquisAT faced difficulties in its attempts to transform working conditions, particularly those related to organizational causes, even though this analysis was expanded to incorporate new instruments and rules derived from activity ergonomics. In other words, the AS of PesquisAT’s productive activity was in a state of crisis (Engeström and Sannino, 2010), requiring a search for new methodologies that could facilitate the implementation of changes.

In CHAT, the relationships among and within the elements of an AS move and evolve historically, stimulating the development of a system in which contradictions, understood as opposed units, forces, or tendencies (Engeström and Sannino, 2011), may manifest.

We suggest that the AS associated with PesquisAT’s productive activity during this pre-CL period mainly focused on dealing with the contradiction between its instruments and its object. Although the instruments allowed for a systemic analysis, they were inadequate for implementing changes in work processes through the effective handling of the complexity of the object. Therefore, the group did not succeed in preventing work-related accidents and occupational diseases or protecting and promote workers’ health.

This was the prevailing context when a member of PesquisAT, participating in scientific events during the 1990s and early 2000s (see Figure 2), witnessed presentations on CHAT and CL that captured his attention. He perceived the potential for such a theory to reconcile diagnosis with transformation of the work environment. Despite the conceptual and institutional advances in workers’ health in Brazil, formative methodologies based on learning theories that can sustain intervention processes and ensure continued development of work processes are rarely applied.

In 2007, a member of PesquisAT met a Brazilian researcher and a Finnish researcher at an event. One outcome of that meeting was a co-authored article by these researchers on the CL method, published in Portuguese in 2011 (Querol et al., 2011), and their development of close ties with PesquisAT. The group felt the need to develop concepts that complemented those derived from ergonomics to advance their understanding of CL and the effectiveness of their interventions. This perception is expressed in the following two comments:

*“From the beginning when I began to read Engeström, what drew my attention to CL*… *was the fact that one did not stop at diagnostics. I mean, it was a process in which diagnostics was part of the changes, and not something in itself, which was something that had always bothered me about ergonomics*… *So, I think that the two things that I liked most were, first, that there was no separation drawn between diagnostics and transformation and, second, that it was something that was very well adapted to organizational transformation.” (Interviewee 1).*

“So, [it was] about a tool; an instrument that was used to draw the interventions, and he (Interviewee 1) saw a connection to an issue that we had been discussing within the group. Because this question of the difficulty of developing the intervention is poorly handled in some of the stuff we write; poorly because we have worried about it for a long time.” (Interviewee 3).

This perceived need for complementary concepts for dealing with the contradictions that PesquisAT was facing at the time led to its adoption of new theoretical frameworks, such as CHAT and CL. Their incorporation consequently led to changes in other elements within its AS.

## The Expansion of Pesquisat’s Learning Platform

The publication of the previously mentioned article on CL in Portuguese helped to consolidate a collaborative partnership between Brazilian and Finnish researchers (Figure 2) and expand an inter-institutional network through exchanges between research teams from the two countries. Notably, the article was published during the time when the PesquisAT group was formulating a thematic project titled “Work accident: From socio-technical analysis toward the social construction of change” (FAPESP proceeding no. 2012/04721-1), which was initially planned for a 4-year period. The induction of new researchers into the group led to the adoption of CHAT and CL within a new theoretical and methodological framework, considered as another critical event. Some opinions about the thematic project are expressed below:

“What they were doing [in Finland] nobody knew here [in Brazil], so I think that it was something to do with the thematic [project], this methodology, a field, an object little known in Brazil, at least within the field of health-related work.” (Interviewee 1).

“[It was] a totally new approach in Brazil that continued because of its innovative character.” (Interviewee 5).

Various dimensions of the thematic project contributed to the widening of the support network, division of labor, and community of PesquisAT’s AS. In 2012 and 2013, Brazilian researchers participated in a summer course on formative interventions offered at the Centre for Research on Activity, Development and Learning (CRADLE) within Helsinki University. In 2015, a Brazilian researcher returned to Finland through a sandwich Ph.D. program supervised by Prof. Emeritus Yrjö Engeström.

In 2012, 2015, and 2017, Finnish researchers went to Brazil and taught courses on CHAT and CL to members of the PesquisAT group, resulting in more qualified Brazilian interventionist researchers. The exchange visits of the research teams can also be considered as critical events influencing the expansion of the network and community and the learning of new activities for training interventionists.

These exchanges contributed to the expanded activities of PesquisAT: the interactions and dynamics experienced by the Brazilian researchers in Finland or by the Finnish researchers during the time they spent in Brazil resulted in a ballast that organized some of the training activities targeting members of PesquisAT. For instance, in 2015, Dr. Laura Seppännen, a Finnish researcher, spent 4 months in Brazil on an exchange visit. During this time, she assisted Brazilian researchers in conducting different CL projects. Moreover, she led CL study seminars for PesquisAT (that continue) and helped to coordinate the submission of research results. Studies that applied the CL method were presented at a session on the theme of CL during the 18th Congress of the Brazilian Ergonomics Association held in 2016. Collaborations and partnerships expanded through participation in scientific events, which provided spaces for debates that fostered learning and disseminated knowledge while also expanding the community.

The exchanges allowed for an increasing trend of continuous participative learning of the group within open, flexible, and institutionalized spaces. This meant that learning was organized in more inclusive spaces that were open to newcomers who wanted to learn this methodology without having to be linked to the proposing institution, while at the same time having institutional recognition. These spaces were reflected in the post-graduate courses, summer courses, and even the study seminars, which were recognized as a form of research action within projects financed by agencies promoting research.

Our analysis of the content of interviews and questionnaires also indicated that the action of directing a course and/or delivering training on formative interventions was also considered a learning strategy, as revealed by the following comment:

“We taught a post-graduate course, I think that it was there and then that I had to stop and study again. I joked about it with the guys, saying ‘every time I grab that book by Jaakko [Virkkunen] on Change Laboratory, it seems that I understand something new.”’ (Interviewee 4).

It is also important to draw attention to the evidence on the practical dimension of learning, namely learning by doing. When the researcher-interventionist trains new interventionists, he or she also learns through the teaching process. It is necessary to prepare and study to deliver a training course, and practical experiences are valuable in the sedimentation of knowledge. This approach is inspired by the critical pedagogy of the acclaimed Brazilian educator, Freire (1998), and our research data are aligned with the founding principles of CHAT. For Freire (1998), teaching, intervening, and learning are part of a single process. In his words:

*“[T]here is no such thing as teaching without research, and research without teaching. One inhabits the body of the other. As I teach, I continue to search and re-search. I teach because I search, because I question, and because I submit myself to questioning. I research because I notice things, take cognizance of them. And in so doing, I intervene. And intervening, I educate and educate myself.”* (Freire, 1998, p. 35).

In a study on the parallels between Vygotsky and Paulo Freire, Alves (2012) raised important considerations that can be better explored through interventionist research and learning processes, whilst also integrating contemporary approaches of expansive learning (Engeström, 1987, 2016). Initially, individuals procure models, theories, and concepts offering potential solutions to meet their needs. They subsequently internalize the knowledge and put it into practice, which leads to the emergence of new creative forms (Engeström, 1999). It is through internalization and externalization that the ascension from the abstract to concrete takes place (Ilyenkov, 1982).

**FIGURE 4:**
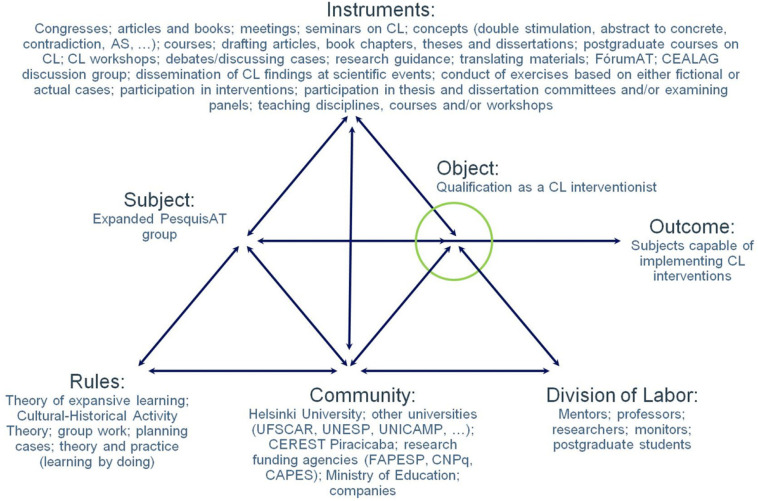
Post-change Laboratory learning platform of the PesquisAT group (2020).

Therefore, we posited that after CL was incorporated into the learning platform (Figure 4), it developed further through the organization of CL study seminars and international cooperation between Brazilian universities and other institutions, for instance CRADLE. Most learning strategies currently used, such as exercises for applying theoretical concepts, already existed. When CHAT and CL principles were adopted by the group, some members took the opportunity to experience them practically and began to apply them consciously and systematically in the planning of training activities (courses, workshops, and training sessions). Examples of these principles include double stimulation, multivoicedness, and “ascending from the abstract to concrete.” Thus, new exercises structured using the double stimulation method were created to present CHAT and CL principles:

“When we started to prepare the post-graduate courses, CL post-graduate courses, we started using its concepts. For instance, with double stimulation, we thought, ‘let’s see such and such case, a concrete case, which is the second stimulus to it?’ Consequently, we began to understand better what that principle was.” (Interviewee 4).

Moreover, actions based on the principles of learning by doing as well as dialogical interactions, internationalization, and mediation were intensified. During the process of planning post-graduate courses, students who wanted to use CL selected actual cases that enabled them to plan how they would conduct negotiations, mirror data collection, choose participants, and complete the cycle of expansive learning implemented during CL sessions. In other words, they collectively planned the entire intervention process in a practical manner. This planning strategy was already being used by Finnish researchers and was adopted at PesquisAT.

One of the procedures applied during the training activities was to divide the students into smaller groups to accomplish the proposed tasks and subsequently to present them to the class for discussion. Each group had a monitor to clarify doubts, facilitate debate, and encourage participation of all group members (multivoicedness). These groups constituted important social spaces where exchanges of experiences and peer learning could occur.

“Group work was an opportunity for learning with other students who knew more about the method.” (Form 23).

*“All of them–post-graduate courses and theoretical seminars–have a fundamental role by providing two pillars: practical and theory pillars. We are making this effort in the seminars, as in the formal post-graduate courses. I think that supervision is necessary; if people want to apply the method, it is not possible to do the course and then start applying it themselves. The idea of partnership involves having somebody who has already done it and is more confident*… *to work in pairs or trios.” (Interviewee 5).*

The sharing of responsibilities among students, researchers, and teachers, with each group learning from the others, also contributes to the successful accomplishment of training activities. Teachers and monitors together planned the event’s activities, determined cases and stimuli to be used, and prepared handouts.

Our analysis of the interviews and forms indicated that compared with earlier courses, recent ones reflected the development of more updated training activities. During the first course, no Brazilian cases were in progress. Although the participants conducted the planning exercise for the entire intervention, they had difficulty moving from the abstract to concrete.

*“I think that it was interesting, since the course in 2012 or in 2015 was taught by them [Finnish researchers] using their examples, articles, cases, everything. I saw in [the course of] 2018*… *people grasped the methodology better than we did when we attended the course [in 2012] with them [Finnish researchers], and I wondered why. Because it was not the first time people [the Brazilian group] had contact with CL. They had seen stuff about CL and also put into practice cases that were already going on. It was different when we started. There were no cases. Everything was theoretical, supposition, right?” (Interviewee 7).*

At the end of the training course of 2012, it was proposed that the participants apply CL concepts and methods to Brazilian cases. This is how the first PesquisAT CL cases were initiated: Piracicaba’s CEREST case (Cerveny et al., 2020), the School Health Centre (Costa et al., 2020), and airport construction (Lopes et al., 2020). The CEREST and School Health Centre projects were proposed as pilot projects during this first training course.

“[Interviewee 5’s] idea of us taking part in [CEREST’s] CL was really interesting! I think that it offered a very rich learning experience, and there were challenges.” (Interviewee 8).

Over time, exchanges also occurred, involving mentoring and the support of Finnish and Brazilian researchers in ongoing research projects in Brazil. The majority of respondents (51.3%) received tutoring for implementing CL. Further, there is evidence that the availability of social spaces for planning and debating are crucial for supporting researchers dealing with the challenges that arise during implementation.

In addition to the use of unfamiliar cases, a practical challenge that constrains learning relates to language: the language of communication in interactions and international cooperation is English. In Brazil, even within academic environments, many researchers are not fluent in English. When the CL training began, there were few publications on CL written in Portuguese. This was a constraint for many PesquisAT members. Therefore, the strategy adopted was to organize initiatives to translate books on CL and didactic materials used by the Finnish researchers for teaching the method in Brazil.

“I think that we would gain a lot in quality if we had a service of specialized simultaneous translation during the exchange events involving professors from Finland.” (Form 10).

The deployment of this strategy is illustrated in the publication in 2015 of a book on CL written by Prof. Emeritus Jaakko Virkkunen and Denise Newnham (Virkkunen and Newnham, 2015) and translated into Portuguese (Figure 2). In a context where very few CL publications were available in Portuguese, publication of this edition enabled the group to master core CL concepts. This book was identified as one of the key didactic resources for learning the method by 29 out of the 39 respondents (74.4%) who completed the questionnaire, followed by articles (identified by 16 respondents [41%]).

To date, the thematic project, which entails a strategy of learning by doing, has led to 10 CL-based formative interventions by PesquisAT members, two of which were conducted in 2014 (Figure 2). The first interventions can be viewed as critical events because the practical application of the method generated the need for learning activities. The interventions reinforced the learn-by-doing principle and promoted dialogical interactions within the group. The flexible and institutionalized spaces that were fostered enabled the formation of expanded teams for practicing and debating the interventions.

“I think it was that; going and doing it, coming back, spending 15 days looking to data and books at the same time. We tried to understand, tried to name what we saw according to the theory. All of that helped us to plan the next step.” (Interviewee 7).

*“So, when one starts working in the field, applying CL*…, *it all begins to sink in.” (Interviewee 4).*

“All of the activities are essential for learning/understanding the method. I consider that experiencing the application of CL (the practice) makes it richer and ratifies the efficiency and efficacy of the method.” (Form 34).

In 2018, we began writing a book reporting on the PesquisAT interventions (Vilela et al., 2020c), which should contribute to disseminating the method and sharing the results of the group’s initiatives within society. These studies have been supported by grants received from different public agencies that promote research, so sharing results is important. The book was translated into English in 2019 and published in 2020. A Portuguese version was released in the same year. The group also considered writing theses and dissertations as well as articles and/or books as a learning strategy. Writing these works requires re-reading the didactic materials and engaging in debates with peers and taking out moments to analyze and reflect on the subject and data.

“The encounter with the idea of learning happens this way, and every time we try to write something down, then it is the moment of stopping to organize thoughts and review.” (Interviewee 3).

When PesquisAT’s first thematic project concluded in 2019, a new partnership was initiated, this time with French researchers who are applying the method of activity ergonomics. The partnership was established through a new thematic project titled “Innovation and Transformation for Prevention Activity of Professional Risks” (ITAPAR). The project was approved and initiated in 2020. It entails the use of the CHAT, CL, and activity ergonomics theoretical interventions to support interventions at different levels (micro, meso, and macro) of activities for the prevention of accidents and occupational diseases. Again, an expansion of the community within the learning platform has occurred.

As discussed in this paper, the expansion of PesquisAT’s learning platform and of its expansive learning activity relating to CL was integral to its trajectory, propelled by a series of contradictions within and among the AS (Figures 3, 4). To deal with the complexity of the new object of the learning platform (Figure 4), the research group had to expand its community by incorporating diverse actors as well as introduce new instruments and rules.

The subjects who engaged in the previous AS of the learning platform did not have CHAT and CL expertise. Therefore, researchers-interventionists needed to be training in these methods to incorporate and support their application within interventions. Accordingly, the group expanded the elements of the AS of its learning platform to establish new processes. It also modified the AS elements of its productive activity that were previously guided mainly by assumptions derived from activity ergonomics. This situation highlights the interdependence of the three levels of activity shown in Figure 1.

We identified the manifestation of a quaternary contradiction associated with CLs developed by PesquisAT between the group’s new methodology (the activity of expansive learning of CL) and its productive activity involving companies and institutions where the interventions were implemented. Participants in CL interventions, namely employees of these companies and institutions, had great difficulty in applying CL concepts to transform their work. This situation led to an innovation: CL training workshops for practitioners, developed by researchers-interventionists. As a result of these workshops, learning has been strengthened and the co-analysis and design of changes in productive activities have evidently improved.

Thus, the interdependence among the three levels of learning activities is once again apparent (Figure 1): the generation of new interventionists led to improved learning processes and to greater sustainability among researcher-interventionists and of the productive activity (Virkkunen and Schaupp, 2011).

Over a period of 8 years, the PesquisAT group has taught hundreds of participants through post-graduate courses and training courses. The theory they impart is complex and requires continuous learning and the use of different strategies, such as seminars, mentoring, and courses.

“I attended the course taught by the Finnish professors, which was very enriching. I am still learning; it is a method that demands a change in references.” (Form 16).

*“It has been a process with ups and downs*, resembling *a histogram. There are moments when I believe that I am learning the method and am aligned with its concepts and applicability. But some readings are more complex and my understanding [of them] is not so fluid.” (Form 15).*

*“When conducting all these strategies we* pursued *a combination of various forms of learning: learning by reading, which was very important, practical learning to address the challenge, planning the CL, and applying the concepts in practice.” (Interviewee 5).*

After 10 formative CL interventions had been conducted by PesquisAT in Brazil, the group realized that continuous formal training with academic courses, tutoring activities, and a permanent debate forum could play an important role in qualifying researchers to implement formative interventions (Vilela et al., 2020b). For instance, participants often participated repeatedly in CL post-graduate courses as students or as mentors. Moreover, we believe that such repetition is justified, as post-graduate participants have different qualifications and zones of proximal development, and in many cases, they are encountering CHAT and CL for the first time.

*“I attended the post-graduate course twice; once during a summer course and later as a regular post-graduate student. The experience of planning and thinking through a case*… *was very important. I consider it to be a constant learning [process] and am now taking part in the planning and implementation of a real CL [intervention]. I am learning a lot more.” (Form 3).*

“[Starting] from the first contact with the research group, summer course, and post-graduate course, the process is continuous; it is lengthy, slow, and expansive.” (Form 12).

In spite of advances, important challenges remain in the implementation of formative interventions relating to productive activity, such as the reluctance of managers within companies where the intervention is being implemented to participate in training sessions. This reluctance usually leads to resistance to solutions formulated during CL sessions (Vilela et al., 2020c).

Our analysis of responses in the questionnaire results (Table 1), categorized as “very expressive” and “expressive,” revealed that the greatest practical challenges in implementing formative interventions were the resistance of senior managers (33.3%), the resistance of middle managers (25.7%) and the negotiation process (25.6%). The resistance of the participating group (23.1%) and fostering agency (20.5%) were identified as moderate difficulties. Some situations were described as being “a little difficult” or “not difficult at all,” namely composing groups to run the process (28.2%) and selecting mirror data (28.2%). The prevalence of “does not apply” answers (ranging between 48.7 and 59%) indicates that most respondents did not participate in actual interventions, even though they may have participated in case planning for post-graduate courses and extracurricular courses.

**TABLE 1:** Difficulties encountered in specific change laboratory-related situations.

Difficulties in specific situations						
Situation	Very Expressive	Expressive	Moderate	Little	None	Does not apply*
Negotiation	23.1%	2.6%	7.7%	12.8%	5.1%	48.7%
Initial agreement	20.5%	0.0%	7.7%	15.4%	5.1%	51.3%
Composition of teams to run the process	5.1%	7.7%	10.3%	20.5%	7.7%	48.7%
Selection of mirror data	0.0%	5.1%	15.4%	20.5%	7.7%	51.3%
Fostering agency	0.0%	7.7%	20.5%	10.3%	2.6%	59.0%
Resistance of the group (participants)	5.1%	10.3%	23.1%	10.3%	2.6%	48.7%
Resistance of middle managers	15.4%	10.3%	15.4%	5.1%	2.6%	51.3%
Resistance of senior managers	23.1%	10.3%	2.6%	2.6%	7.7%	53.9%
Difficulties in designing a new activity system	5.1%	7.7%	15.4%	0.0%	12.8%	59.0%

*Participants were instructed to enter “does not apply” if they had not been in such a situation.

Our results show that even though PesquisAT’s learning platform is critical and has led to better results in CL sessions, the group needs to extend its training of the subjects involved in the AS for the productive activity, notably managers and technical staff, to develop the intervention and achieve organizational learning. We believe that this process can be agreed upon during negotiations and that it will contribute to expansive learning after the interventionist projects end.

Our experience shows that interventionist methodologies have to be adapted to the specific cultural-historical context of each country as well as the characteristics of the targeted activity. In Brazil, authoritarianism is a preeminent principle within work management models, which had a bearing on the difficulties noted by respondents in negotiating and implementing innovations conceived during the CL session.

We understand that factors such as hierarchical relationships and social recognition of the problem operating in a more localized sphere determine the levels of engagement of the different actors in work transformations. Thus, participants’ resistance is always contingent on the local reality, the organization’s interest, and the team’s competences in conducting the intervention in a specific context (Vilela et al., 2020b).

In spite of the difficulties faced in implementing solutions relating to the productive activities within a CL intervention, PesquisAT has progressed, as reflected by the results of the learning platform. Below, we present our findings on knowledge acquisition, abilities and attitudes, and behavioral changes in participants involved with the learning platform after undergoing training, and the results in the long run, drawing on the Kirkpatrick and Kayser-Kirkpatrick (2014) model.

## Participants’ Perceptions of Learning During the CL Training Activities

The majority of participants who completed the questionnaire on CL training activities were women (77%), with 50% of the respondents aged 36 years or younger. This concentration of women may reflect the commonly observed composition of post-graduate programs in Brazil (CAPES and Ministério da Educação Brasil, 2018).

The breakdown of the professional occupations of the participants was as follows: professors (26%), psychologists (15%), physiotherapists (10%), engineers (8%), and other professions, such as physicians, sociologists, justice attorneys, nutritionists, occupational therapists, nurses, and lawyers (41%). The majority had attained an advanced level of education, notably a master’s degree (51.3%), a Ph.D. (23.0%), a graduate degree (15.4%), or a post-doctoral degree (10.3%), and 74.4% of the participants were conducting research activities. The implementation of this method, which is linked to master’s and doctoral programs, and the role of researchers in conducting the interventions probably accounts for the high educational status of participants (84.6% had at least a master’s degree).

These results reveal a challenge for the group regarding the transformation of the method into a non-academic tool that can be used routinely by staff within NGOs and/or interventionist organizations that deal with developing work and environment (e.g., Fundacentro and CEREST). Another challenge relates to the training of the individuals who will use the tool, given that it has theoretical and methodological underpinnings, the understanding of which requires a certain degree of academic training, as pointed out by one of the respondents:

“I don’t know how, but it needs to be better publicized. It needs to appear in non-scientific publications as well, and it needs to enter the academic courses of the various professions dealing with health and safety and work.” (Form 8).

The majority of respondents who completed the questionnaire evaluated current knowledge about the CL method as being “reasonable” (43.6%), followed by evaluations of “good” or “very good” (41.0%), with 15.4% of respondents considering it to be weak. A total of 46.2% of the respondents had participated in a CL intervention, 33.3% frequently participated in CL seminars, and the remaining 66.7% did not participate or participated sporadically. In addition, participation in interventions (30.8%), courses (20.5%), and group discussions (17.9%) were identified by the respondents as being the most important mode of learning.

These results reveal the importance of the collective, a learning-by-doing approach, and a continuous learning process. Evidently, the group’s strategy of involving students in processes of data collection, planning, and participation in interventions is important for their learning. Initially, this strategy of promoting students’ participation was aimed at supporting researchers, but over time, it became deliberate and focused on learning. However, despite its acknowledged importance, the vast majority of respondents did not participate in any interventions and do not participate in CL seminars or do so sporadically. This factor may have influenced the evaluations of a majority of respondents (43.6%) that current knowledge about the method was “reasonable.” In general, the participants’ perceptions of the learning process were positive: 41.0% evaluated their knowledge as being “good” or “very good” and only 15.4% evaluated it as being “weak.” In addition, 92.3% of the respondents considered what they had learned from participating in CL activities to be useful or very useful. In response to a question asking respondents to assess the use of a CL concept or tool in their professional activities, 53.8% stated that they use some concept that they learned in their daily lives. Among the most used concepts mentioned by respondents were the AS model, double stimulation, historical analysis, and contradictions, as revealed in the following response:

*“Not right now, but until July this year I was mediating a project.*…*In this project, which lasted two and a half years, the concepts and tools of the CL helped me a lot to understand the different contexts of the maternity hospitals where I worked. I shared them with other mediators and even with the coordinators of the project, and soon a publication on the project will come out, which will include this report.” (Form 9).*

Table 2 presents the results for mastery of concepts under the categories “good,” “very good,” or “excellent.” The majority of the participants reported mastery of the concepts of historical analysis of the activity (64.1%), the AS as a unit of analysis (64.1%), the object (59.0%), and the expansive learning cycle (53.8%). The concepts perceived by respondents as more challenging under the categories “bad” and “terrible” were the germ cell concept (48.7%) and the quaternary contradiction (38.5%).

**TABLE 2:** Mastery of the Cultural-Historical Activity Theory and Change Laboratory concepts.

Mastery of Concepts						
Concept	Excellent	Very good	Good	Tolerable	Bad	Terrible
Primary contradiction	5.1%	10.3%	28.2%	33.3%	12.8%	10.3%
Secondary contradiction	7.7%	12.8%	23.1%	30.8%	15.4%	10.3%
Tertiary contradiction	2.6%	15.4%	18.0%	33.3%	15.4%	15.4%
Quaternary contradiction	2.6%	12.8%	18.0%	28.2%	23.1%	15.4%
Activity system as the unit of analysis	10.3%	18.0%	35.9%	28.2%	5.1%	2.6%
Double stimulation	10.3%	10.3%	25.6%	35.9%	12.8%	5.1%
Transformative agency	10.3%	5.1%	30.8%	25.6%	23.1%	5.1%
Germ cell	2.6%	7.7%	23.1%	18.0%	35.9%	12.8%
Zone of proximal development	7.7%	12.8%	15.4%	33.3%	20.5%	10.3%
Historical analysis of activity	15.4%	15.4%	33.3%	30.8%	5.1%	0.0%
Object	5.1%	12.8%	41.0%	35.9%	5.1%	0.0%
Cycle of expansive learning	7.7%	12.8%	33.3%	35.9%	7.7%	2.6%
Networks of activity systems	7.7%	12.8%	28.2%	35.9%	12.8%	2.6%
Ascending from the abstract to the concrete	2.6%	5.1%	33.3%	30.8%	20.5%	7.7%

Our analysis of the data allowed us to reflect on aspects of the CL learning process that require improvement. We understand that the concepts indicated as being more difficult to grasp by the students are also among the least encountered concepts during the courses, possibly because the team still experienced difficulty constructing mediating artifacts for such concepts in classroom situations. However, their understanding deepens in a real-life situation when following a case, and some of the respondents had not yet participated in such situations. Nevertheless, it may be necessary to deploy new learning strategies for the CL seminars, such as readings about the subjects, exercises that reinforce these concepts, and organizing debates.

A question on what improvements could be incorporated into this process was posed with the aim of improving the learning process. The main suggestions were participation in practical sessions, the use of practical examples, discussion of one concept per class, production of videos, increased dissemination of the method, and group discussion of papers. Some of these recommendations have already being incorporated into the CL post-graduate program that will be taught this year.

## Final Considerations

In this paper we investigated how should interventionist researchers could be prepared to perform formative interventions. We addressed this question through an analysis of the emergence and development of a learning platform to foster interventionists for conducting formative interventions.

Our findings indicate that learning formative methodologies are essentially collective, necessitating platforms for collective debate and reflection (Haapasaari and Kerosuo, 2015). Actions that are interactive and dialogical, namely those that apply dialogical tools (e.g., group discussions and mentoring) rather than those that seek to unite unidirectional concepts exposition (e.g., lectures and books), can promote a more effective training process. Mentoring demonstrates that effective learning of the CL method entails interactions among individuals with different levels of experience and knowledge in which those with more experience act as learning facilitators. This finding resonates with Vygotsky’s (1978) concept of the zone of proximal development, which he defined as the distance between current development, determined by an individual’s ability to solve problems, and this individual’s potential level determined through collaborations with more capable colleagues.

The findings suggest that the learning process for interventionists appeared to follow the logic of expansive learning (Engeström and Sannino, 2010). As Freire (1998) observed, the elaboration of knowledge, the content of which is historically produced as the accumulated knowledge within a culture, implies following a path of creating a distance from practice in order to “admire it,” seeing it from different angles, and seeing oneself reflected in it within a process mediated by dialogue with others. A point repeatedly made in the interviews and questionnaire responses was that theory only becomes clear when it is used to analyze and intervene in reality. This practical learning requires the creation of spaces for follow up and practical experimentation (e.g., follow-up interventions). When knowledge is submitted to practice, it ascends from the abstract to concrete. This aspect has been recognized by the group, which encouraged the students to participate in interventions and incorporate real case studies within post-graduate courses.

Finally, this study suggests that the provision of training in the use of the platform can be facilitated through the application of the double stimulation method (Vygotsky, 1978; Sannino and Laitinen, 2015). In post-graduate teaching, participants are assigned tasks during courses and debates (e.g., seminars), for instance, texts or case studies that serve as the initial stimulus as well as analytical concepts to analyze the cases, which serve as a second stimulus. Consequently, participants are able to assimilate the concepts.

In this article, we have examined the learning process applied by PesquisAT and have discussed the importance of creating a learning platform for producing qualified interventionists and promoting learning during sessions that continues after the life of a project. Despite advances made by the group, there are still important practical challenges entailed in conducting formative interventions. Among the many challenges faced by the group, the following are critical: (1) difficulty negotiating with managers and persuading them to agree to the intervention, (2) participants’ resistance, and (3) the reluctance of senior managers to accept innovations developed by workers during the CL intervention. Such challenges highlight the need to develop other learning platforms in parallel with CL. The functioning and structure of these other platforms in ways that support participants’ learning outside of the sessions requires further exploration.

Traditional research focuses on producing generalizations through statistical analysis of correlations between cause and effect. This type of generalization is called by Davydov (1990) abstract empirical generalizations, which are useful when the relationship between variables and factors are relatively stable. However, this type of generalization is limited when the empirical conditions vary or the object of research is under construction or does not yet exist. In this study we wanted to produce knowledge about a constantly changing object of research - the learning process of PesquisAT group. Thus, we adopt an approach called theoretical genetic generalization, which is aimed at revealing the genetic roots of a phenomenon and the function of the studied system.

The key contribution of this study is in showing four key principles to foster learning a formative intervention method: (1) promoting dialogues and exchange of experiences, (2) creating environments for continuous learning and permanent discussion (seminars and post-graduate courses and the use of communication technologies), (3) creating spaces for experimentation and the practical application of concepts (case studies and participation in interventions), and (4) the use of the double stimulation method during training programs.

The main limitations of this study are those related to the question of whether there was something that took place in PesquisAT group that could be generalized to other groups interested in learning formative interventions. The validity of this study does not rely on statistical representation but on the historical validity. Although probably there is no other research group in the world that is exactly the same as PesquisAT, there are groups facing the same or similar contradictions faced by the group. The contribution of our study is to show how these contradictions were solved and how the group managed to learn a formative intervention method. In order to increase the validity of the findings more research is needed from other groups.

## Data Availability Statement

The datasets presented in this article are not readily available because the raw data identify the participants. This dataset can be accessed only by the researchers of this study. Requests to access the datasets should be directed to ML, lopes_manoela@yahoo.com.br.

## Ethics Statement

The studies involving human participants were reviewed and approved by the research Ethics Committee of the School of Public Health at the University of São Paulo under CAAE protocol 36516620.6.0000.5421. The patients/participants provided their written informed consent to participate in this study.

## Author Contributions

All the authors contributed to the conception and design of the study, wrote sections of the manuscript, contributed to manuscript revisions, and read and approved the submitted version. ML, AS-M, and VG organized the database and performed the analysis. ML wrote the first draft of the manuscript.

## Conflict of Interest

The authors declare that the research was conducted in the absence of any commercial or financial relationships that could be construed as a potential conflict of interest.

## Footnotes

1 SUS is an acronym for the Portuguese term for the Brazilian public health system, whose main principles are universality, equity, and integrality. This system aims to promote interorganizational action among different services that impact on the health and quality of life of individuals.

2 Innovation and Transformation for Prevention Activity of Professional Risks (ITAPAR) is a thematic project conducted by members of PesquisAT.

3 CEREST is a branch of SUS that performs surveillance actions relating to workers’ health.

4 Fundacentro is a research institution that focuses on the production of applied knowledge that supports public policies aimed at promoting safe, healthy, and productive work.

5 FAPESP is a renowned public institution that promotes academic research.

## References

[B1] Alves S. M. (2012). *Freire e Vigotski um Diálogo Entre a Pedagogia Freiriana e a Psicologia Histórico Cultural.* Chapecó: Editora Argos.

[B2] Arkoudis S. Watty K. Baik C. Yu X. Borland H. Chang S. (2013). Finding common ground: enhancing interaction between domestic and international students in higher education. *Teach. High. Educ.* 18:3. 10.1080/13562517.2012.719156

[B3] Blackmore C. (2007). What kinds of knowledge, knowing and learning are required for addressing resource dilemmas? A theoretical overview. *Environ. Sci. Policy* 10:6. 10.1016/j.envsci.2007.02.007

[B4] Bodrožiæ Z. (2008). *Post-Industrial Intervention: An Activity-Theoretical Expedition Tracing the Proximal Development of Forms of Conducting Interventions.* Helsinki: Helsingfors Universitet.

[B5] Bodrožiæ Z. Adler P. S. (2018). The evolution of management models: a neo-Schumpeterian theory. *Adm. Sci. Q.* 63:1. 10.1177/0001839217704811

[B6] CAPES and Ministério da Educação, Brasil (2018). *Discentes da Pós-Graduação Stricto Sensu do Brasil.* Available online at: https://dadosabertos.capes.gov.br/dataset/2017-2020-discentes-da-pos-graduacao-stricto-sensu-do-brasil/resource/37fde9f4-bb94-4806-85d4-5d744f7f76ef (accessed September 23, 2020).

[B7] Carlfjord S. Roback K. Nilsen P. (2017). Five years’ experience of an annual course on implementation science: an evaluation among course participants. *Implement. Sci.* 12:101. 10.1186/s13012-017-0618-4 28768546 PMC5541724

[B8] Ceccim R. B. (2005). Educação Permanente em Saúde: desafio ambicioso e necessário. *Interface Comunic. Saúde. Educ.* 9:16. 10.1590/S1414-32832005000100013

[B9] Ceccim R. B. Feria A. A. (2009). “Educação permanente em saúde,” in *Dicionário da Educação Profissional em Saúde*, eds Ferreira L. de Almeida Barbosa J. S. da Cruz M. M. (Rio de Janeiro: Fundação Oswaldo Cruz. Escola Politécnica de Saúde Joaquim Venâncio), 162–167.

[B10] Cerveny G. C. O. Coluci M. Z. O. Mendes R. W. B. Vilela R. A. G. (2020). “The clash between new and old models of surveillance system: a case study of Change Laboratory in a workers’ health reference Center,” in *Collaborative Development for the Prevention of Occupational Accidents and Diseases*, eds Vilela R. A. G. Querol M. A. P. Beltrán-Hurtado S. L. Cerveny G. C. O. Lopes M. G. R. (Cham: Springer International Publishing), 191–204. 10.1007/978-3-030-24420-0_13

[B11] Cole M. (1998). *Cultural Psychology: A Once and Future Discipline.* Cambridge, MA: Harvard University Press.

[B12] Costa S. V. C. Silva-Macaia A. A. Querol M. A. P. Vilela R. A. G. (2020). “Shared construction of change scenarios for academic activities: the case of a school of public health and its school health center,” in *Collaborative Development for the Prevention of Occupational Accidents and Diseases*, eds Vilela R. A. G. Querol M. A. P. Beltrán-Hurtado S. L. Cerveny G. C. O. Lopes M. G. R. (Cham: Springer International Publishing), 145–157. 10.1007/978-3-030-24420-0_10

[B13] Davydov V. V. (1990). *Types of Generalization in Instruction: Logical and Psychological Problems in Structuring School Curricula.* Reston, VA: National Council of Teachers of Mathematics.

[B14] Denzin N. (1970). *Research Act in Sociology: A Theoretical Introduction to Sociological Methods.* London: Aldine Publishing Company.

[B15] Dias E. C. (1994). *A Atenção à Saúde dos Trabalhadores no Setor Saúde (SUS), no Brasil: Realidade, fantasia ou utopia.* Dissertation, Faculdade de Ciências Médicas da Universidade Estadual de Campinas, Campinas.

[B16] Engeström Y. (1987). *Learning by Expanding: An Activity-Theoretical Approach to Developmental Research.* Helsinki: Orienta-Konsultit.

[B17] Engeström Y. (1999). “Activity theory and individual and social transformation,” in *Learning in Doing: Social, Cognitive, and Computational Perspectives. Perspectives on Activity Theory*, eds Engeström Y. Miettinen R. Punamäki R. L. (Cambridge: Cambridge University Press), 19–38. 10.1017/CBO9780511812774.003

[B18] Engeström Y. (2001). Expansive learning at work: toward an activity-theoretical reconceptualization. *J. Educ. Work* 14:1. 10.1080/13639080020028747

[B19] Engeström Y. (2007). “Putting vygotsky to work: the change laboratory as an application of double stimulation,” in *The Cambridge Companion to Vygotsky*, eds Daniels H. Cole M. Wertsch J. V. (New York: Cambridge University Press), 363–425. 10.1017/CCOL0521831040.015

[B20] Engeström Y. (2011). From design experiments to formative interventions. *Theory Psychol.* 21:5. 10.1177/0959354311419252

[B21] Engeström Y. (2016). *Aprendizagem Expansiva.* Campinas: Pontes Editores.

[B22] Engeström Y. Pihlaja J. Helle M. Virkkunen J. Poikela R. (1996). The Change Laboratory as a tool for transforming work. *Lifelong Learn. Europe* 1 10–17.

[B23] Engeström Y. Sannino A. (2010). Studies of expansive learning: foundations, findings and future challenges. *Educ. Res. Rev.* 5:1. 10.1016/j.edurev.2009.12.002

[B24] Engeström Y. Sannino A. (2011). Discursive manifestations of contradictions in organizational change efforts: a methodological framework. *J. Organ. Chan. Manag.* 24:3. 10.1108/09534811111132758

[B25] Engeström Y. Sannino A. Virkkunen J. (2014). On the methodological demands of formative interventions. *Mind. Cult. Act.* 21:2. 10.1080/10749039.2014.891868

[B26] Freire P. (1998). *Pedagogy of Freedom: Ethics, Democracy, and Civic Courage.* Lanham, MD: Rowman & Littlefield Publishers.

[B27] Guérin F. Kerguelen A. Laville A. Daniellou F. Duraffourg J. (2004). *Compreender o trabalho para transformá-lo: A prática da Ergonomia.* São Paulo: Edgard Blücher.

[B28] Haapasaari A. Engeström Y. Kerosuo H. (2014). The emergence of learners’ transformative agency in a Change Laboratory intervention. *J. Educ. Work* 29 232–262. 10.1080/13639080.2014.900168

[B29] Haapasaari A. Engeström Y. Kerosuo H. (2018). From initiatives to employee-driven innovations. *Eur. J. Innov. Manag.* 21:2. 10.1108/EJIM-09-2016-0085

[B30] Haapasaari A. Kerosuo H. (2015). Transformative agency: the challenges of sustainability in a long chain of double stimulation. *Learn. Cult. Soc. Interact.* 4 37–47. 10.1016/j.lcsi.2014.07.006

[B31] Han H. Boulay D. (2013). Reflections and future prospects for evaluation in human resource development. *New Horizons Adult Educ. Hum. Resour. Dev.* 25:2. 10.1002/nha.20013

[B32] Hurtado S. L. B. Vilela R. A. G. Almeida I. M. Jackson Filho J. M. Querol M. A. P. Simões R. R. (2020). “Contributions from the Change Laboratory to the analysis and prevention of accidents’ model,” in *Collaborative Development for the Prevention of Occupational Accidents and Diseases*, eds Vilela R. A. G. Querol M. A. P. Beltrán-Hurtado S. L. Cerveny G. C. O. Lopes M. G. R. (Cham: Springer International Publishing), 207–224. 10.1007/978-3-030-24420-0_14

[B33] Ilyenkov E. V. (1982). *The Dialectics of the Abstract and the Concrete in Marx’s Capital.* Moscow: Progress Publishers.

[B34] Kirkpatrick D. L. Kirkpatrick J. D. (2006). *Evaluating Training Programs. The Four Levels.* San Francisco, CA: Berrett-Koehler Publishers.

[B35] Kirkpatrick J. Kayser-Kirkpatrick W. (2014). *The Kirkpatrick Four Levels: A Fresh Look after 55 Years.* Newnan, GA: Kirkpatrick Partners.

[B36] Laitinen A. Sannino A. Engeström Y. (2016). From controlled experiments to formative interventions in studies of agency: methodological considerations. *Educação* 39:14. 10.15448/1981-2582.2016.s.24321

[B37] Le Masson P. Weil B. Hatchuel A. (2009). “Platforms for the design of platforms: collaborating in the unknown,” in *Platforms, Markets and Innovation*, ed. Gawer A. (Cheltenham: Edward Elgar Publishing), 273–305.

[B38] Leontiev A. N. (1978). *Activity, Consciousness, and Personality.* Englewood Cliffs, NJ: Prentice-Hall.

[B39] Leontiev A. N. (1981). *Problems of the Development of the Mind.* Moscow: Progress Publishers.

[B40] Lima F. P. L. Dias A. V. C. (2020). “Work and health and contemporary capitalism: economics as a social disease,” in *Collaborative Development for the Prevention of Occupational Accidents and Diseases*, eds Vilela R. A. G. Querol M. A. P. Beltrán-Hurtado S. L. Cerveny G. C. O. Lopes M. G. R. (Cham: Springer International Publishing), 29–48. 10.1007/978-3-030-24420-0_3

[B41] Lopes M. G. R. Vilela R. A. G. Querol M. A. P. (2018). Agency for a systemic comprehension of work accidents and organizational anomalies. *Trabalho Educ. Saúde* 16:2. 10.1590/1981-7746-sol00128

[B42] Lopes M. G. R. Vilela R. A. G. Querol M. A. P. Almeida I. M. (2020). “Challenges to Change Laboratory learning in a dynamic and complex civil construction project,” in *Collaborative Development for the Prevention of Occupational Accidents and Diseases*, eds Vilela R. A. G. Querol M. A. P. Beltrán-Hurtado S. L. Cerveny G. C. O. Lopes M. G. R. (Cham: Springer International Publishing), 131–144. 10.1007/978-3-030-24420-0_9

[B43] Minayo-Gomez C. Thedim-Costa S. M. (1997). A construção do campo da saúde do trabalhador: percurso e dilema. *Cadernos de Saúde Pública* 13(Suppl. 2) S21–S32. 10.1590/S0102-311X1997000600003 10886935

[B44] Ministério da Saúde, Brasil (2012). *Portaria no̱ 1.823, de 23 de agosto de 2012. Institui a Política Nacional de Saúde do Trabalhador e da Trabalhadora. Saúde legis: sistema de legislação da saúde.* Available online at: http://bvsms.saude.gov.br/bvs/saudelegis/gm/2012/prt1823_23_08_2012.html (accessed December 14, 2018).

[B45] Ministério da Saúde, Brasil (2014). *Educação Permanente em Saúde.* Brasília: Ministério da Saúde, Brasil.

[B46] Muller J. Z. (2018). *The Tyranny of Metrics.* Princeton, NJ: Princeton University Press.

[B47] Paavola S. Lipponen L. Hakkarainen K. (2004). Models of innovative knowledge communities and three metaphors of learning. *Rev. Educ. Res.* 74:4. 10.3102/00346543074004557

[B48] Praslova L. (2010). Adaptation of Kirkpatrick’s four level model of training criteria to assessment of learning outcomes and program evaluation in higher education. *Educ. Assess. Evaluation. Account.* 22:3. 10.1007/S11092-010-9098-7

[B49] Querol M. A. P. Hurtado S. L. B. SanJosé C. M. Espinosa I. V. Guzman W. C. Casals E. T. (2019). Formative interventions in education and learning at work: application of the Change Laboratory in Ibero-America. *Rev. Intern. Educ. Aprendizaje* 7:2.

[B50] Querol M. A. P. Jackson Filho J. M. Cassandre M. P. (2011). Change laboratory: uma proposta metodológica para pesquisa e desenvolvimento da aprendizagem organizacional. *Admin. Ensino Pesquisa* 12:4. 10.13058/raep.2011.v12n4.143

[B51] Reeves S. Peller J. Goldman J. Kitto S. (2013). Ethnography in qualitative educational research. AMEE Guide No. 80. *Med. Teach.* 35 e1365–e1379. 10.3109/0142159X.2013.804977 23808715

[B52] Röling N. (1994). “Platforms for decision making about ecosystems,” in *The Future of the Land: Mobilizing and Integrating Knowledge for Land Use Options*, eds Fresco L. O. Stroosnijder L. Bouma J. van Keulen H. (Chichester: John Wiley and Sons), 385–393.

[B53] Röling N. G. (1992). The emergence of knowledge systems thinking. A changing perception of relationships among innovation, knowledge process and configuration. *Knowl. Policy* 5 42–64. 10.1007/BF02692791

[B54] Sannino A. Engeström Y. (2017). Co-generation of societally impactful knowledge in Change Laboratories. *Manage. Learn.* 48:1. 10.1177/1350507616671285

[B55] Sannino A. Engeström Y. Lemos M. (2016). Formative interventions for expansive learning and transformative agency. *J. Learn. Sci.* 25:4. 10.1080/10508406.2016.1204547

[B56] Sannino A. Laitinen A. (2015). Double stimulation in the waiting experiment: testing a Vygotskian model of the emergence of volitional action. *Learn. Cult. Soc. Inter.* 4 4–18. 10.1016/j.lcsi.2014.07.002 26318436

[B57] Sewell W. H. Jr. (1996). Historical events as transformations of structures: inventing revolution at the Bastille. *Theor. Soc.* 25:6. 10.1007/BF00159818

[B58] Söderlund L. L. Madson M. B. Rubak S. Nilsen P. (2011). A systematic review of motivational interviewing training for general health care practitioners. *Patient. Educ. Couns.* 84:1. 10.1016/j.pec.2010.06.025 20667432

[B59] Taras V. Caprar D. V. Rottig D. Sarala R. M. Zakaria N. Zhao F. (2013). A global classroom? Evaluating the effectiveness of global virtual collaboration as a teaching tool in management education. *Acad. Manag. Learn. Educ.* 12:3. 10.5465/amle.2012.0195

[B60] Vänninen I. Pereira-Querol M. Engeström Y. (2015). Generating transformative agency among horticultural producers: an activity-theoretical approach to transforming integrated pest management. *Agr. Syst.* 139 38–49. 10.1016/j.agsy.2015.06.003

[B61] Vilela R. A. G. Almeida I. M. Mendes R. W. B. (2012). Da vigilância para prevenção de acidentes de trabalho: contribuição da ergonomia da atividade. *Ciência Saúde Coletiva* 17:10. 10.1590/S1413-81232012001000029 23099767

[B62] Vilela R. A. G. Jackson Filho J. M. Querol M. A. P. Gemma S. F. B. Takahashi M. A. C. Gomes M. H. P. (2018). A expansão do objeto da vigilância em acidente do trabalho: história e desafios de um centro de referência em busca da prevenção. *Ciência Saúde Coletiva* 23:9. 10.1590/1413-81232018239.21952016 30281742

[B63] Vilela R. A. G. Querol M. A. P. Almeida I. M. Jackson Filho J. M. (2020a). “Workers’ health: from diagnosis to formative intervention,” in *Collaborative Development for the Prevention of Occupational Accidents and Diseases*, eds Vilela R. A. G. Querol M. A. P. Beltrán-Hurtado S. L. Cerveny G. C. O. Lopes M. G. R. (Cham: Springer International Publishing), 3–11. 10.1007/978-3-030-24420-0_1

[B64] Vilela R. A. G. Querol M. A. P. Hurtado S. L. B. Cerveny G. C. O. Lopes M. G. R. (2020b). *Collaborative Development for the Prevention of Occupational Accidents and Diseases.* Cham: Springer International Publishing. 10.1007/978-3-030-24420-0

[B65] Vilela R. A. G. Querol M. A. P. Seppänen L. Lima F. P. A. Mendes R. W. B. Lopes M. G. R. (2014). “Work ergonomic analysis and Change Laboratory: similarities and complementarities between interventionist methods,” in *Proceedings of the AHFE* (Berlin: Springer), 19–23.

[B66] Vilela R. A. G. Querol M. A. P. Silva-Macaia A. A. Hurtado S. L. B. (2020c). “Learning in and from Change Laboratory interventions for developing workers’ health in Brazil,” in *Collaborative Development for the Prevention of Occupational Accidents and Diseases*, eds Vilela R. A. G. Querol M. A. P. Beltrán-Hurtado S. L. Cerveny G. C. O. Lopes M. G. R. (Cham: Springer International Publishing), 225–253. 10.1007/978-3-030-24420-0_15

[B67] Virkkunen J. Newnham D. S. (2013). *The Change Laboratory: A Tool for Collaborative Development of Work and Education.* Rotterdam: Sense Publishers. 10.1007/978-94-6209-326-3

[B68] Virkkunen J. Newnham D. S. (2015). *O Laboratório de Mudança: uma Ferramenta de Desenvolvimento Colaborativo Para o Trabalho e a Educação.* Belo Horizonte: Fabrefactum Editora.

[B69] Virkkunen J. Schaupp M. (2011). From change to development: expanding the concept of intervention. *Theory Psychol.* 21:5. 10.1177/0959354311417486

[B70] Vygotsky L. S. (1978). *Mind in Society: The Development of Higher Psychological Processes.* Cambridge, MA: Harvard University Press.

[B71] Vygotsky L. S. (1998). *Pensamento e Linguagem.* São Paulo: Martins Fontes.

[B72] Wals A. E. J. (2007). *Social Learning Towards a Sustainable World: Principles, Perspectives, and Praxis.* Wageningen: Wageningen Academic Publishers. 10.3920/978-90-8686-594-9

